# The Fitting and Furnishing of Wards and Operating Theatres

**Published:** 1906-09-29

**Authors:** 


### THE FITTING AND FURNISHING OF WARDS AND OPERATING THEATRES.

### WARD LOCKERS, TABLES, ETC.

The material of which ward lockers, tables, dressing-trolleys, instrument cupboards, etc., are composed is now very largely metal, in some cases aluminium, in others steel, painted with fire-stoved enamel paint, and with glass and opalite shelves and tops. On the other hand in several of the leading London hospitals hard wood is preferred for lockers, cupboards, and tables, the matter being occasionally varied by the use of hard wood bodies to the lockers, and the legs of the tables, with opalite or glass tiles for the tops and covers. Aluminium is also being introduced in place of the glass shelves, where steel frames are employed, because the aluminium shelf is easily kept clean and bright. It is lighter than the glass shelf it displaces, it is equally as aseptic, and is not liable to breakage. On the other hand, aluminium is very easily attacked by alkalis. The idea underlying the use of glass for shelves, and the tops of ward lockers is, of course, the fact that glass cannot easily harbour dirt or germs if properly dusted, but the glass slabs that are employed are very expensive. For the dressing-trolleys light steel frames painted with stove-fired enamel, the frames being made with recesses to hold the solution bottles, trays, etc., the whole trolley being supported on substantial rubber castors, with a motion in all directions, so that the trolley is moved very easily, and without noise from bed to bed, appears to be a favourite form. In some hospitals *x*-ray apparatus is mounted upon a trolley, something similar to the dressing-trolley though larger, and is wheeled to the patient's bedside. Instrument cupboards are nearlyl all of steel stove-enamelled with glass sides, tops and bottoms, and glass shelves, the doors of the cupboards being arranged to close quite air-tight, and in closing to slightly compress the air within the cupboard.

### Ventilation of the Wards.

The plenum system has not yet been much adopted for the ventilation of the wards in the leading hospitals, with a few notable exceptions, of which the Birmingham General Hospital and the Victoria Hospital at Belfast are the most important. In the wards of these hospitals there are no fire grates. The ventilating current enters the ward under some of the windows in each ward, passing up through a duct forming a lining to the wall underneath the window, and it passes out by a similar number of ducts situated usually under the beds, in the immediate neighbourhood of the entry ducts. In the wards of these hospitals windows cannot be opened. The hospital staff speak very highly of it, nurses praising the lessened labour in the matter of dusting, and in keeping up fires at night, but neither nurses nor doctors care to have it in their own quarters, and doctors almost uniformly complain that something has been taken out of the atmosphere. As was mentioned by the present writer in an article in The Hospital, he makes out that plenum air has been deprived, in a great many cases, of a certain quantity of oxygen. In the great majority of hospitals ventilation is entirely by windows, of which there are always plenty, portions of the windows in many hospitals being arranged on the louvre system, so that the entering air is largely directed towards the ceiling. The window ventilation is assisted by that of the chimneys, and by the arrangements for heating the air, by the aid of radiators placed in front of inlet gratings, in outside walls. In spite of all improvements there is no ventilation equal to that of the chimney, if it is properly arranged; and when fires are alight, the fire itself has some effect in destroying any germs that may reach it; while, as remarked in another article, the light rays emitted by a cheerful fire, such as Englishmen love, have also a very useful office. With some forms of ward-stoves, those in particular made by Messrs. Doulton, with an upcast flue, ventilation is assisted by a duct placed in the neighbourhood of the flue, where it enters the floor above, the ventilating air current passing in to this ventilating duct, by holes forming concentric circles round the flue. The presence of the flue heats the air in its neighbourhood, and materially assists the air current. The ventilation of the sanitary blocks of each ward is very largely on the "cross-current" system. There are windows on each side of the passage leading into the ventilating block, and in a great many directions of the wind, a current of air passes across from one window to the other, producing what engineers call an injector effect, the result being the extraction of air from the lavatories, etc., and its being carried off through the window of the passage on the lee-side.

### Lifts.

There are two forms of lifts in use in the leading hospitals, worked respectively by hydraulic and by electrical power. Hydraulic power is the older, but the modern electrically driven and electrically controlled lift is so much more convenient that it is rapidly taking its place. In the electric lift, in place of pulling a rope and there being a jerk at starting and at stopping, the control is entirely by a switch on the cage, the lift starting and stopping without jerk. In addition arrangements can be made for controlling the lift from any floor. That is to say, by pushing a button upon any floor, the lift, if not in use, comes to that floor. Lift gates cannot be opened when electrically controlled, unless the cage is opposite a floor.

### Lighting the Wards.

Electricity is employed almost universally for lighting the wards of the principal hospitals, the incandescent lamp of 16 and 32 c.p. being generally employed. The Nernst lamp, however, is gradually making its way, because of the whiter light it gives, and the smaller quantity of electricity it requires. For lowering the light in the wards at night two principal systems are used. In one, three lamps are held under a conical shade, all three giving their full light during the evening, and being arranged by means of a simple switch to give only a red light at night. The other method is, all the lamps are switched off except one which is screened from the body of the ward. The double filament lamps now on the market, in which a small filament takes the place of the ordinary filament at night, do not seem to have yet been tried.

### Telephones.

Telephonic communication between the wards and the different administrative departments are entirely on what is called the inter-communication system, in which each room to be communicated with has a telephone apparatus, and a multiple switch. Each room has its number, and to communicate with it the arm of the multiple switch in any other room is turned to the number of that room, the usual telephone operations being then proceeded with, the switch arm being returned to the number marked O, when the communication is finished. The Berliner Company and Messrs. Ericcsson make apparatus in which communication is not cut off with any number if its switch is not returned to zero.

